# Responses of Soil Fungal Community Structure, Co-Occurrence Networks, and Functions to Different Oak-Dominated Mixed Plantations

**DOI:** 10.3390/plants15091399

**Published:** 2026-05-02

**Authors:** Yanfang Wang, Xiaoqiu Yuan, Zhichao Li, Zhengyang Yan, Yage Li, Ling Liu

**Affiliations:** 1 College of Agriculture, Henan University of Science and Technology, Luoyang 471023, China; wyfll1977@126.com (Y.W.);; 2 Luoyang Forestry Ecological Construction and Development Center, Luoyang 471026, China

**Keywords:** co-occurrence network, monoculture, mixed plantations, *Quercus variabilis*, soil fungal community

## Abstract

*Quercus variabilis* is one of the primary species for plantation regeneration across China’s warm-temperate and subtropical zones. However, its long-term monoculture leads to ecosystem instability. Soil fungi are essential for nutrient cycling and ecosystem functioning, yet their responses to oak-dominated mixed plantations remain insufficiently understood. This study investigated the soil fungal communities among *Q. variabilis* monoculture (QV), mixed plantations of *Q. variabilis* and *Platycladus orientalis* (PO), *Q. variabilis* and *Pinus tabuliformis* (PT), and *Q. variabilis*, *P. orientalis* and *P. tabuliformis* (PPQ). The results showed that PO and PPQ plantations contained significantly higher concentrations of SOC, TN, and TP compared to QV monoculture. Ascomycota and Basidiomycota were identified as the dominant fungal phyla across four plantation types, with PO exhibiting the highest relative abundance of Ascomycota (60.85%) and fungal alpha diversity. The soil fungal communities across all plantations were predominantly saprotrophic, followed by mixotrophic modes. The relative abundance of saprotrophic fungi was significantly greater in the mixed plantations, peaking in PO at 44.69%. The soil fungal communities in both PO and PPQ plantations exhibited higher network interaction density. The SOC, TN, TP, water content, zinc, and β-glucosidase activity served as key environmental drivers of fungal community composition. Overall, the mixed plantation of *Q. variabilis* and *P. orientalis* most effectively improved soil fertility, enhanced fungal diversity, and increased network complexity, suggesting its potential as a sustainable afforestation strategy for oak-dominated ecosystems in the low hilly regions of western Henan. However, these findings are based on a single sampling period, and long-term monitoring is required to confirm its sustained ecological benefits.

## 1. Introduction

Soil microorganisms act as fundamental ecosystem engineers, driving nutrient cycling, forming soil structure, and underpinning overall ecosystem productivity and stability [1,2]. Fungi play vital roles in the decomposition of organic matter, nutrient uptake for plants, and the stabilization of soil structure. In forest ecosystems, fungi constitute the dominant soil biota and are key drivers of organic matter decomposition, plant nutrient acquisition, and soil structural maintenance [3,4]. The composition and functional attributes of soil fungal communities are strongly influenced by tree species and management practices [5]. Variations in litter inputs and root exudation among tree species selectively shape fungal assemblages, thereby indirectly affecting nutrient cycling processes and tree growth [6]. Mixed-species plantations have been shown to enhance microbial biomass and diversity while reshaping the structure of soil microbial communities [7]. Furthermore, the structure and function of the soil fungal population are also governed by environmental factors and inter-fungal interactions [8].

Linking microbial community structure with ecosystem functioning is essential for understanding soil processes and supporting sustainable soil and plant management [9]. Recent advances in high-throughput sequencing technologies have greatly improved the characterization of fungal community composition and functional organization in soils [10]. Soil fungal functional guilds are now widely recognized as sensitive bioindicators of soil health due to their capacity to reflect environmental fluctuations. Distinct fungal guilds play critical roles in plant nutrient acquisition, organic matter turnover, and disease regulation [11]. Mycorrhizal fungi, for example, establish mutualistic associations with more than 80% of terrestrial plant roots, thereby enhancing nutrient uptake and promoting plant growth [12,13]. In contrast, saprotrophic fungi function as primary decomposers through the enzymatic degradation of dead organic substrates, including woody debris and leaf litter, facilitating nutrient release and sustaining ecosystem nutrient cycling [14]. Pathogenic fungi, however, regulate plant population dynamics and influence ecosystem diversity via host-specific infections, ultimately reshaping biodiversity patterns and driving community succession [15,16,17].

Microbial co-occurrence network analysis maps the complex associations of soil microorganisms, which can reflect the interaction of soil microbes and potential ecological functions. This method is also important for identifying key species and predicting microbial community responses to environmental changes [18,19]. Numerous studies have demonstrated that soil physicochemical properties, nutrient availability, plant communities, and environmental disturbance together influence the topology and stability of soil microbial co-occurrence networks. Li et al. [20] reported that legume plantations exhibited a more intricate interaction network and higher soil nutrients compared to non-legume plantations. According to Wang et al. [21], plantation age and soil compartment significantly shaped soil microbial networks in *Eucalyptus* plantations, and rhizosphere soil microbial communities were closely linked to total soil nitrogen and microbial biomass. Beyond these relationships, their key finding was that network complexity plays a more vital role than microbial diversity or the presence of nutrient functional genes in driving soil carbon and nitrogen cycling processes. Microbial co-occurrence analysis across diverse forest types provides critical insights into the structure and ecological functions of soil fungal communities, forming a scientific basis for developing effective soil management and sustainable forestry practices [22].

*Quercus variabilis*, one of the most widely distributed oak species in China, mainly grows on well-drained slopes in warm temperate and subtropical regions [23]. *Q. variabilis* has the advantages of strong adaptability, fast growth rate, and high-quality timber and is the main species of afforestation in barren hillsides for conserving soil [24]. Numerous studies have been conducted on monoculture oak plantations, including management practices, growth dynamics, ecosystem stability, anatomical traits, and ecosystem service functions [25,26]. These studies suggest that monoculture often leads to ecological problems such as soil degradation, biodiversity loss, and pest outbreaks, which hinder the sustainable development of forest ecosystems. Several studies have shown that mixed oak forests can enhance soil fertility and ecosystem stability [27,28,29]. Although some studies have examined oak growth, soil nutrients, and insect resistance in mixed oak forests [30,31], the responses of soil physicochemical properties, fungal community assembly, and co-occurrence network patterns to different mixed oak plantations remain insufficiently understood. This study investigated soil samples from four plantation types: *Q. variabilis* monoculture (QV), mixed plantations of *Q. variabilis* and *Platycladus orientalis* (PO), mixed plantations of *Q. variabilis* and *Pinus tabuliformis* (PT), and mixed plantations of *Q. variabilis*, *P. orientalis* and *P. tabuliformis* (PPQ). We characterized the diversity and composition of soil fungal communities and their relationships with key soil factors across the varying mixed QV plantations. Our findings provide new insights into the ecological mechanisms underlying mixed plantation systems and offer a scientific basis for designing effective afforestation and sustainable management strategies for mixed oak forests.

## 2. Results

### 2.1. Soil Physicochemical Properties and Enzyme Activity

As shown in Table 1, the QV monoculture exhibited significantly higher soil BD than other mixed plantations (*p* < 0.05), whereas SWC did not differ significantly among the four plantation types. All of the plantations exhibited slightly alkaline soil, and the QV monoculture had higher pH values than the mixed plantations. Soil nutrient contents were generally enhanced in mixed plantations. Compared with QV, SOC increased by 18.32% (PO) and 19.45% (PPQ), while PT showed a moderate increase of 6.25%. Similarly, TN increased by 8.90% (PO) and 19.18% (PPQ), whereas it slightly decreased by 3.42% in PT. TP content was significantly higher only in PO, with an increase of 20.83% relative to QV, while PT and PPQ showed no significant changes. Soil TK increased by 11.85% (PO), 9.93% (PT), and 5.11% (PPQ) relative to QV (*p* < 0.05). These increases suggest enhanced organic matter input and more active carbon and nutrient cycling in mixed plantations. In addition, the contents of Fe, Mg, and Zn were higher in both PO and PT plantations than in the QV monoculture. Enzyme activities exhibited differential responses among plantation types. URE, involved in nitrogen mineralization, showed no significant differences, indicating relatively stable nitrogen transformation rates. In contrast, CAT, associated with microbial oxidative stress regulation, was 31.58% higher in PO than in QV. Carbon-acquiring enzymes showed contrasting trends. BG, which catalyzes the degradation of labile carbon substrates, increased significantly by 58.42% (PO), 38.44% (PT), and 19.53% (PPQ) compared with QV, indicating enhanced carbon turnover. Conversely, CBH, associated with the breakdown of more recalcitrant carbon fractions, decreased by 17.68% (PO), 20.87% (PT), and 12.13% (PPQ), suggesting a shift in microbial carbon utilization strategies. Nitrogen- and phosphorus-acquiring enzymes were also enhanced. NAG, involved in chitin degradation and nitrogen release, increased by 20.72% (PO), 61.86% (PT), and 43.54% (PPQ), while AKP, which mediates organic phosphorus mineralization, increased by 37.58% (PO), 27.47% (PT), and 10.70% (PPQ) relative to QV. These patterns suggest enhanced microbial nutrient acquisition and tighter coupling of carbon–nitrogen–phosphorus cycling in mixed plantations.

### 2.2. Soil Fungal Abundance and Community Diversity

**Figure 1:**
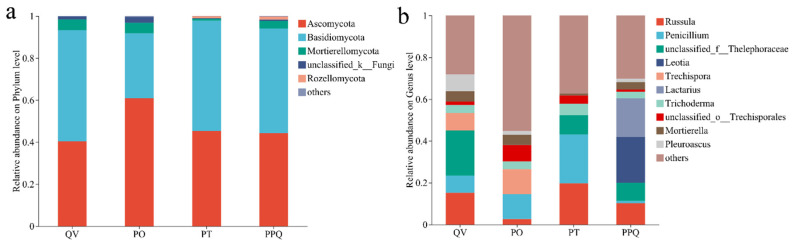
Soil fungal community composition at the phylum (**a**) and genus (**b**) levels in monoculture and mixed *Q. variabilis* plantations.

At the phylum level, the soil fungal communities across QV, PO, PT, and PPQ plantations were consistently dominated by Ascomycota and Basidiomycota, with relatively low abundances of Rozellomycota, unclassified_k__Fungi, and other minor phyla (Figure 1a). The combined relative abundance of Ascomycota and Basidiomycota reached 93.27%, 91.86%, 97.83%, and 94.16% in QV, PO, PT, and PPQ, respectively. Notably, the relative abundance of Ascomycota in PO (60.85%) was higher than that in other plantations. In contrast, QV exhibited a higher relative abundance of Basidiomycota (52.97%). Additionally, Mortierellomycota was more abundant in the QV plantation (5.12%) than in the PO, PT, and PPQ plantations (Figure 1a).

At the genus level, the ten most prevalent taxa accounted for 71.90%, 44.82%, 62.67%, and 69.77% of the total sequences in the QV, PO, PT, and PPQ plantations, respectively. Dominant genera differed among plantation types, with unclassified_*f__Thelephoraceae* (21.85%) predominating in QV, *Trechispora* (11.95%) in PO, *Russula* (19.65%) together with *Penicillium* (23.43%) in PT, and *Leotia* (22.02%) along with *Lactarius* (18.52%) in PPQ (Figure 1b), showing significant variation in relative abundance across plantations (*p* < 0.05).

### 2.3. Alpha Diversity of Soil Fungal Communities

**Figure 2:**
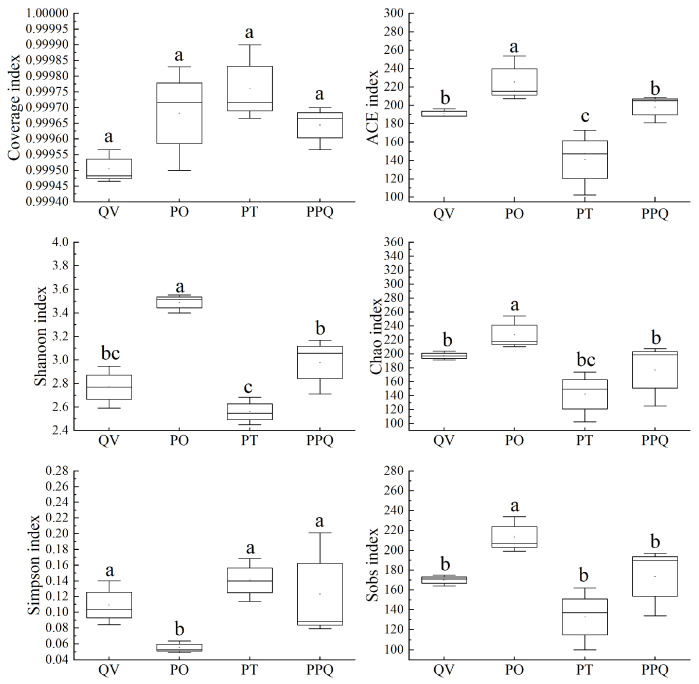
Alpha diversity of soil fungal communities in monoculture and mixed *Q. variabilis* plantations. Different letters indicate significant differences among plantation types (*p* < 0.05).

Goods coverage indices ranged from 0.9995 to 0.9999, confirming that the sequencing depth adequately captured the full spectrum of soil fungal communities in the QV, PO, PT, and PPQ plantations. The PO plantation exhibited the highest Ace (225.39), Shannon (3.48), Chao (227.50), and Sobs (213.30) indices, which were significantly higher than those of the other plantation types (*p* < 0.05), indicating the greatest fungal community richness and diversity (Figure 2). In contrast, the PT plantation showed the highest Simpson index, which was significantly higher than those of the other plantation types (*p* < 0.05), suggesting a higher degree of species dominance and lower community evenness within its fungal assemblage.

### 2.4. Beta Diversity of Soil Fungal Communities

**Figure 3:**
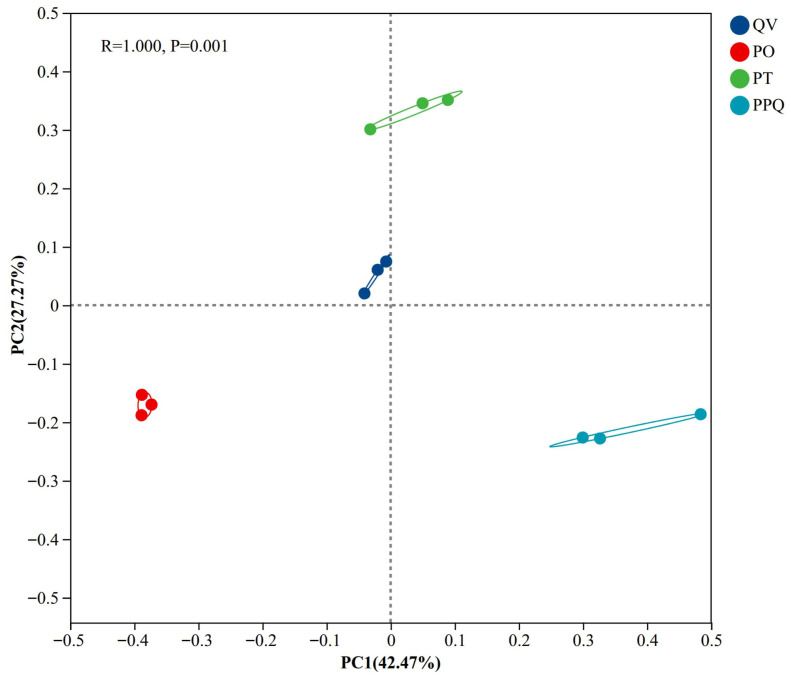
Principal coordinate analysis of soil fungal communities in monoculture and mixed *Q. variabilis* plantations.

The ANOSIM test yielded an R value of 1.0 and a *p*-value of 0.001, indicating significant differences in soil fungal communities among plantation types (Figure 3). In the principal coordinate analysis, PC1 and PC2 explained 69.74% of the total variance. Soil fungal communities from different artificial mixed oak forests showed various distributions along the PC1 and PC2 principal coordinates. The PO, PT, and PPQ clusters were in the lower left, upper right, and lower right quadrants of the principal coordinate plot, respectively. Each cluster had its own set of soil fungal community traits. The QV cluster occupied the upper left quadrant, with relatively small and concentrated values for PC1 and PC2, indicating a more homogeneous fungal community structure.

**Figure 4:**
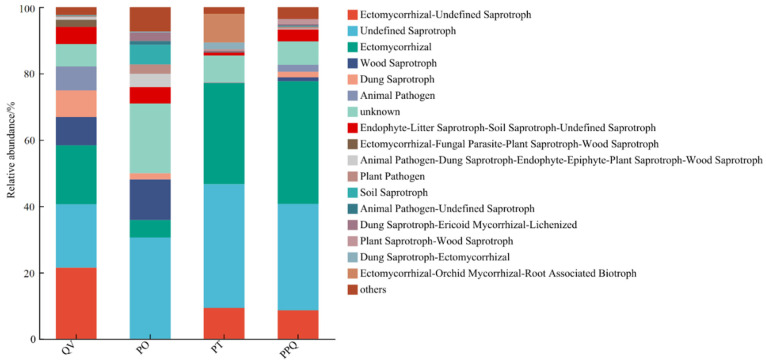
Functional prediction of soil fungi using FUNGuild in monoculture and mixed *Q. variabilis* plantations.

Based on the FUNGuild database, the trophic modes of soil fungal communities in QV, PO, PT, and PPQ plantations were classified into four primary categories: saprotroph, pathotroph, symbiotroph, and mixotroph (Figure 4, Table 2). The mixotroph category included five subtypes: saprotroph–symbiotroph, pathotroph–saprotroph–symbiotroph, pathotroph–symbiotroph, pathogen–saprotroph–symbiotroph, and pathotroph–saprotroph. Across all forest types, saprotroph was the most common trophic mode, with relative abundances of 35.85% in QV, 44.69% in PO, 37.96% in PT, and 36.50% in PPQ. Mixotrophs represented the second most abundant category, accounting for 29.70%, 12.65%, 10.70%, and 13.14% of the fungal communities in QV, PO, PT, and PPQ, respectively.

Functional groups within fungal communities differed across forest types (Table 2). Soils in the QV plantation were enriched with dung saprotrophs and ectomycorrhizal–undefined saprotrophs, with relative abundances of 8.02% and 21.5%, respectively, which were significantly higher than those in other plantations (*p* < 0.05). Similarly, the PO plantation showed significantly greater relative abundances of soil saprotrophs (5.93%) and animal pathogen–undefined saprotrophs (1.12%). The PT plantation was distinguished by a significantly higher abundance of ectomycorrhizal–orchid mycorrhizal–root associated biotrophs (8.68%). The PPQ plantation hosted significantly more plant saprotrophs–wood saprotrophs (1.55%) and ectomycorrhizal fungi (36.98%) than the other plantations (*p* < 0.05).

### 2.5. Soil Fungal Networks and Topological Properties

**Figure 5:**
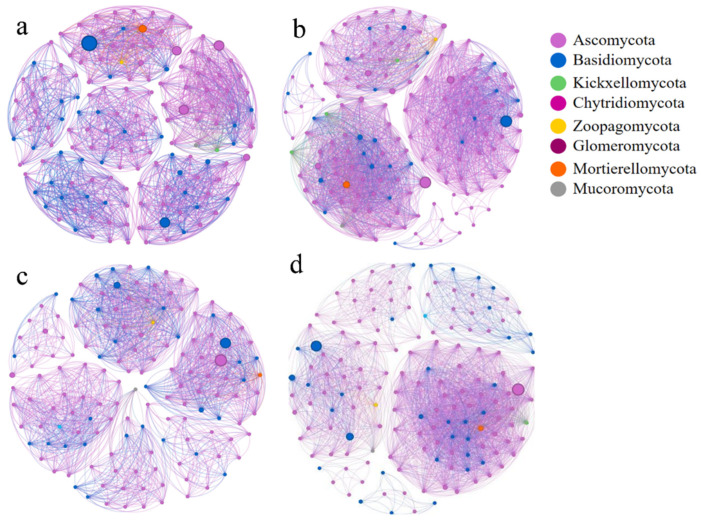
Co-occurrence networks of soil fungal communities at the genus level in *Q. variabilis* monoculture plantation (**a**), mixed *Q. variabilis* and *P. orientalis* plantation (**b**), mixed *Q. variabilis* and *P. tabulaeformis* plantation (**c**), and mixed *Q. variabilis*, *P. orientalis*, and *P. tabulaeformis* plantation (**d**) based on Spearman’s correlation coefficient. Node size reflects the degree of connectivity for each genus, whereas node color indicates its corresponding module classification. Edge width corresponds to the magnitude of the Spearman correlation coefficient (|ρ|) between paired genera.

The complexity of soil fungal communities in monoculture and mixed *Q. variabilis* plantations was investigated by constructing co-occurrence networks (Figure 5). Soil fungal networks in the four plantations exhibited pronounced modularity, with modularity indices varying across plantations. The QV plantation soil fungal network showed the highest modularity index (0.823), while the PPQ plantation soil fungal network had the lowest (0.524). The PPQ plantation soil fungal network had the most edges (2890), followed by the PO plantation (2852). The QV plantation soil fungal network had the fewest edges (1782), which means that the PPQ plantation soil fungal community had more interactions. The proportion of positively correlated edges was generally higher than that of negatively correlated edges across all networks, with PPQ exhibiting the highest proportion of positive correlations (90.45%), indicating more frequent positive interactions among its fungal communities. Additionally, the networks of different mixed oak plantations showed distinct mean degrees and graph densities. The mean degrees and graph densities of fungal networks in PO and PPQ soils were 19.013 and 0.128, 19.267 and 0.129, respectively, both higher than those in QV and PT plantations. This indicates denser and more interconnected fungal networks in PO and PPQ plantations, reflecting efficient connectivity and information transfer among nodes within the network, which may contribute to enhancing overall community stability and function. In contrast, the QV plantation exhibited lower mean degree and network density in its soil fungal network, at 12.041 and 0.082, respectively.

### 2.6. Effects of Soil Physicochemical Properties and Enzyme Activity on Fungal Community Structure

**Figure 6:**
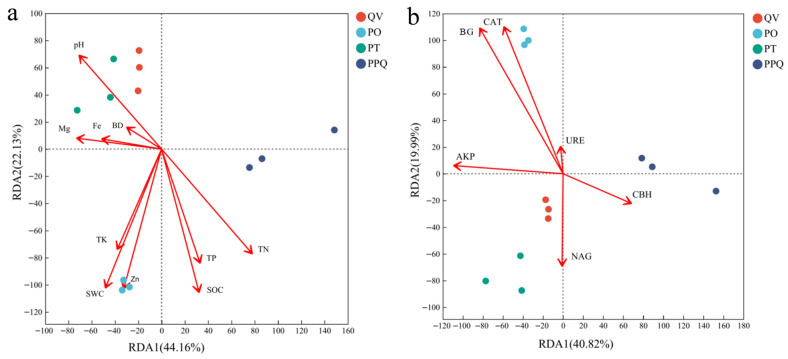
Relationships between soil physicochemical properties (**a**) and enzyme activities (**b**) and fungal community structure based on redundancy analysis (RDA).

As illustrated in Figure 6, the first two RDA axes accounted for 66.29% and 60.81% of the variation associated with soil physicochemical properties and enzyme activities, respectively. The composition of soil fungal communities across plantation types showed differential associations with environmental variables. Among the measured factors, SOC, TN, TP, SWC, pH, Zn, and BG were closely related to shifts in fungal community structure. In PO and PPQ plantations, these indicators were positively associated with fungal community composition. Specifically in the PO, SOC (r = 0.85, *p* < 0.01), TN (r = 0.80, *p* < 0.05), TP (r = 0.78, *p* < 0.05), SWC (r = 0.76, *p* < 0.05), Zn (r = 0.70, *p* < 0.05), and BG (r = 0.85, *p* < 0.05) showed the strongest positive associations. In contrast, the PT and QV demonstrated negative associations with these environmental variables. In the QV, SOC (r = −0.72, *p* < 0.01), TN (r = −0.65, *p* < 0.05), TP (r = −0.70, *p* < 0.01), SWC (r = −0.68, *p* < 0.05), Zn (r = −0.50, *p* < 0.05), BG (r = −0.90, *p* < 0.01), NAG (r = −0.55, *p* < 0.05), and AKP (r = −0.65, *p* < 0.05) were negatively correlated with fungal community structure, suggesting potential constraints on fungal assemblages under this plantation type.

## 3. Discussion

### 3.1. Effects of Plantation Type on the Composition and Diversity of the Soil Fungal Community

Soil fungal communities are important drivers of forest ecosystem function, and their composition and diversity are sensitive indicators reflecting the dynamics of fungal communities and important biological indicators for assessing soil quality [32,33]. In this study, we identified Ascomycota and Basidiomycota as the two predominant phyla across all plantation types. Previous studies have consistently demonstrated that Ascomycota and Basidiomycota represent prevalent and characteristic terrestrial fungal groups, frequently serving as the dominant fungal components in the soils of deciduous, mixed evergreen-deciduous, and evergreen broadleaf forests [34,35,36]. Ascomycota, consisting largely of saprophytes and parasites, are particularly effective in decomposing resistant organic compounds like lignin and plant fibers. Similarly, Basidiomycota contribute significantly to organic matter decomposition and nutrient cycling [37,38]. Our results revealed marked disparities in the proportions of major fungal taxa among the plantation types. The following factors may explain these variations: First, the quantity, chemical composition, and quality of litter inputs (e.g., leaves and branches) differed among the different tree species [39,40]. For instance, Coniferous litter (e.g., *P. tabuliformis* and *P. orientalis*) is typically rich in recalcitrant compounds such as waxes, tannins, and resins, leading to slower decomposition rates. Such conditions favor the taxa of Ascomycota, which specifically decompose resistant organic materials such as lignin and tannins. In contrast, broadleaf litter (e.g., *Q. variabilis*) contains higher proportions of cellulose and hemicellulose, making it more labile and potentially supporting the growth of saprotrophic Ascomycota and saprotrophic Basidiomycota. The result is supported by the higher relative abundance of Ascomycota in the three mixed oak–coniferous plantations compared to the QV monoculture plantation. Our findings also showed that the PO plantation exhibited a higher relative abundance of Ascomycota compared to other mixed plantations. The result suggests that mixed plantations of *Q. variabilis* and *P. orientalis* can increase the relative abundance of Ascomycota and improve soil nutrients. Consistent with this observation, the PO plantation showed higher SOC, TN, and TP contents than the QV monoculture plantation. In the PO plantation, the Ascomycota in the soil were predominantly composed of saprophytic fungal communities, which efficiently decomposed litter and converted it into stable soil nutrients, establishing a healthy soil fertility cycle. Second, afforestation-induced alterations in the soil microenvironment significantly influence fungal communities [41]. Changes in the soil physicochemical properties (e.g., pH, water content, and nutrient availability) drive compositional shifts in fungal assemblages as they adapt to the modified environment [42,43]. Third, differences in root exudates among tree species influence the activity and composition of rhizosphere microorganisms [44,45]. In our study, both *Q. variabilis* and *P. tabulaeformis* are ectomycorrhizal plants, and the compounds secreted from their roots specifically stimulate and recruit ectomycorrhizal fungi. In contrast, *P. orientalis* is a host of arbuscular mycorrhizae and can form symbionts with these endomycorrhizal fungi. In QV monoculture plantation, PT mixed plantation, and PPQ mixed plantation, ectomycorrhizal fungi (many belonging to the Basidiomycota) associated with the roots of *Q. variabilis* and *P. tabulaeformis* may dominate, thereby relatively suppressing the abundance of Ascomycota. In this study, the *Q. variabilis* plantation was characterized by a significantly elevated relative abundance of Basidiomycota.

The alpha diversity of soil microorganisms exhibits the richness and evenness of species within a microbial community. Higher community diversity and more complex community structure may be associated with greater potential stability in soil ecosystems and stronger resilience to external disturbances [46,47]. Previous studies have shown that mixed oak plantations can significantly alter litter composition and decomposition rates, consequently modifying the composition and function of soil microbial communities [48,49]. In our study, the soil fungal communities in the PO exhibited the highest Ace, Shannon, Chao, and Sobs indices, while PT showed the highest Simpson index. The result indicates that the fungal community of PO possesses the highest richness and diversity, whereas the PT plantation is dominated by a larger proportion of dominant species. These results suggest that the mixed planting of *Q. variabilis* and *P. orientalis* has advantages in cultivating soil fungal communities with rich species, complex structure and balanced functions.

The observed differences in fungal community structure among plantation types reflect not only variations in tree species but also differences in environmental factors. Principal coordinate analysis (PCoA) revealed a more clustered distribution of fungal communities in the QV plantation, whereas those in the PO, PT, and PPQ mixed plantations showed greater dispersion. This pattern indicates higher community homogeneity in the *Q. variabilis* monoculture plantation and greater heterogeneity in the mixed plantations. The divergence of soil fungal community may relate to the changes in soil environment induced by tree species and the interactions between plants and microorganisms [50]. The higher soil fungal community similarity in the monoculture forest may be attributed to uniform litter composition and similar root exudates, resulting in limited micro-environmental variation. In contrast, the diverse litter inputs and root exudates in mixed plantations likely created more heterogeneous soil microenvironments, thereby fostering divergent fungal community assemblages. Overall, these results suggest that variations in tree species composition and associated environmental factors jointly influence soil fungal community composition and diversity, with mixed plantations potentially supporting more heterogeneous and diverse fungal communities.

### 3.2. Functional Group Analysis of Soil Fungal Communities in Monoculture and Mixed Q. variabilis Plantations

Different afforestation patterns have significant effects on fungal functional groups, and soil fungi in turn can adapt to environmental changes through different nutritional modes. In this study, saprophytic fungi were the dominant trophic mode among soil fungi in all plantations, followed by mixotrophic fungi, consistent with findings by Zhao et al. [51]. The relative abundance of saprophytic fungi in PO soils reached 44.69%, higher than in other forest types. Studies indicate that saprophytic fungi are closely associated with elevated soil nutrient levels [52,53]. In this study, PO plantation showed higher soil nutrients, which means that *Q. variabilis* and *P. orientalis* mixed plantations improved soil fertility and promoted the growth and reproduction of soil saprophytic fungi. Additionally, the relative abundance of ectomycorrhizal fungi was higher in the PT and PPQ plantations compared to other plantation types, primarily attributed to the presence of *Q. variabilis* and *P. tabuliformis*, which are known for forming strong ectomycorrhizal associations. The specific signaling molecules and carbon sources released by their roots specifically attract and support the colonization and growth of ectomycorrhizal fungi [54].

Co-occurrence network analysis has emerged as a valuable complement to conventional diversity analysis and offers critical insights into the complex interrelationships within fungal communities [55]. In our study, QV exhibited the highest network modularity index, suggesting a more compartmentalized network structure. In contrast, PO and PPQ showed higher network interaction densities, which were associated with greater connectivity among fungal taxa. This structural divergence may be linked to differences in resource availability: the monoculture system (QV) provides relatively homogeneous resources, such as uniform litter and root exudates, potentially resulting in discrete fungal modules that occupy specific resource niches. Mixed plantations (PO and PPQ), by contrast, offer heterogeneous resources, including diverse litter inputs and varied root exudates, creating more differentiated ecological niches. These conditions are associated with denser and more interconnected networks, in which fungal species may coexist and interact more intensively. Overall, these patterns suggest that mixed plantations may support more complex fungal networks and potentially higher functional redundancy, which could contribute to greater ecosystem resilience, although causal relationships cannot be inferred from correlation alone.

### 3.3. Impact of Soil Factors on Soil Fungal Communities in Monoculture and Mixed Q. variabilis Plantations

Different afforestation types cause changes in soil properties, which are associated with variations in the structure and function of soil microbial communities [56,57]. This study demonstrated that SOC, TN, TP, SWC, pH, Zn, and BG were the principal factors correlated with soil fungal communities, aligning with previous studies [2,58]. Lozano et al. [59] found that vegetation types influenced the microbial community composition, which was indirectly mediated by soil physicochemical properties, such as SWC and nutrient availability. Similarly, Kutos et al. [60] found that fungal communities are closely related to spatial heterogeneity in soil characteristics within woodland ecosystems.

RDA analysis indicated that the soil physicochemical characteristics and enzymatic activities of different oak-mixed plantations were differently associated with soil fungal communities. In PO plantations, SOC, TN, TP, pH, SWC, Zn, and BG were positively associated with fungal community composition, potentially reflecting favorable soil conditions and active carbon cycling. In contrast, in QV monocultures, these factors were negatively associated with fungal communities, possibly reflecting functional constraints related to monoculture systems, such as limited litter diversity or C/N imbalance, which can alter microbial interactions and nutrient dynamics. The comparatively simpler fungal networks and lower alpha diversity in QV plantations are consistent with these patterns.

Soil enzyme activities were also associated with nutrient elements, indicating potential interactions between microbial processes and soil nutrient availability. For example, BG activity showed positive associations with SOC and TN, suggesting that carbon-degrading enzyme activity may be linked to substrate availability and nutrient cycling processes. These patterns are consistent with the relatively simpler network structures and lower alpha diversity observed in the QV plantation. The more heterogeneous resource inputs in mixed plantations (e.g., diverse litter and root exudates) may contribute to improved soil conditions and support more complex fungal community structures. Xu et al. [19] showed that continuous monoculture can lead to microbial imbalance and functional decline. In QV monoculture systems, uniform litter inputs may contribute to C/N imbalance or the accumulation of inhibitory compounds, potentially disrupting interactions between soil properties and microbial communities. In addition, the relatively lower stability of monoculture systems may increase their sensitivity to environmental stress, further influencing fungal community structure [34,49]. These results suggest that differences in soil properties, enzyme activities, and resource inputs among plantation types may play an important role in shaping soil fungal communities, although the underlying mechanisms require further investigation. A limitation of this study is that soil sampling was performed only once (in August). Fungal communities in temperate forests are known to exhibit strong seasonal dynamics, with significant shifts in community composition, diversity, and functional group abundances across seasons. A single-time-point sampling provides only a snapshot of the fungal community and may not capture the full annual variation in trophic modes or treatment effects. Therefore, our findings should be interpreted as reflecting the summer condition only. Future research incorporating multi-season sampling and long-term monitoring would be essential to better understand the stability, resilience, and ecological significance of fungal community dynamics in mixed plantation systems.

## 4. Materials and Methods

### 4.1. Study Area

This study was conducted at the state-owned DahuLing Forest Farm (34°03′–34°14′ N, 112°25′–112°33′ E), situated in the low hilly region of western Henan Province, China. The forest farm represents the largest state-owned public welfare plantation in Henan Province and maintains an overall forest coverage of approximately 90%. The study site is characterized by a warm temperate continental monsoon climate, with an average annual temperature of 14 °C and annual precipitation of around 690 mm. The soil is predominantly classified as Haplic Luvisols, derived from sandstone and shale parent materials. The major tree species found in the forest farm include *Q. variabilis*, *P. tabulaeformis*, *Albizia julibrissin*, *Gleditsia sinensis*, *P. orientalis*, and *Robinia pseudoacacia*. Shrubs include *Tamarix ramosissima*, *Ziziphus jujuba var. spinosa*, *Lespedeza bicolor*, and *Vitex negundo*. The herbaceous layer is composed of *Kalimeris indica*, *Artemisia argyi*, *Artemisia selengensis*, *Artemisia annua*, *Artemisia scoparia*, *Adenophora triphylla*, *Carex lanceolata*, *Brachypodium sylvaticum*, *Artemisia capillaris*, *Carex umbrosa*, *Xanthium strumarium*, *Setaria viridis*, *Oxalis corniculata*, *Matricaria chamomilla*, *Chrysanthemum indicum*, *Clematis heracleifolia*, *Potentilla chinensis*, *Hemerocallis fulva*, and *Ophiopogon japonicus.*

### 4.2. Experimental Design and Soil Sampling

Field surveys were conducted in August 2023 in collaboration with staff from the DahuLing Forest Farm. We selected four plantation types established in 1993 and growing under similar site conditions, including comparable slope, altitude, and soil characteristics. The plantations included *Q. variabilis* monoculture (QV), mixed plantation of *Q. variabilis* and *P. orientalis* (PO), mixed plantation of *Q. variabilis* and *P. tabuliformis* (PT), and mixed plantation of *Q. variabilis*, *P. orientalis*, and *P. tabuliformis* (PPQ). The composition ratios of the mixed plantations were as follows: QV and PO at a ratio of 6:4; QV and PT at a ratio of 6:4; and QV, PT, and PO at a ratio of 6:2:2. All plantations received standard management, including weeding and tending during the first three years after afforestation, with no significant disturbances thereafter. For each plantation type, three replicate plots (20 m × 20 m) were established, totaling 12 plots. A buffer distance of at least 20 m was upheld between neighboring plots. Soil samples were collected from the 0–20 cm layer using a composite sampling strategy. In each plot, five subsamples were collected following an S-shaped pattern to ensure spatial representativeness and then combined into one composite sample. In mixed plantations, sampling points were randomly distributed across each plot to capture the integrated effects of coexisting tree species. The same sampling protocol was applied to the QV monoculture. To obtain representative material, five subsamples from each plot were homogenized and transported to the laboratory under cooled conditions. Prior to analysis, visible roots and coarse debris were removed and the processed soil was subsequently sieved (2 mm) and partitioned according to analytical requirements. Subsamples intended for molecular analysis were preserved at −80 °C before DNA extraction, whereas those designated for enzyme assays were maintained at 4 °C. The remaining fraction was used for determining pH and nutrient-related properties, including carbon, nitrogen, and phosphorus. In addition, undisturbed soil cores were obtained from the 0–20 cm layer using the cutting ring approach to evaluate bulk density and soil moisture content.

### 4.3. Determination of Soil Physicochemical Properties

Fresh soil samples were oven-dried at 105 °C to a constant mass for gravimetric estimation of soil water content (SWC). Soil pH was assessed potentiometrically in a 1:2.5 (*w*/*v*) soil–water suspension using a calibrated pH meter (Mettler Toledo, Columbus, OH, USA). Bulk density (BD) was evaluated based on intact cores obtained via the cutting ring approach. Soil organic carbon (SOC) was quantified through potassium dichromate oxidation with external heating [61], whereas total nitrogen (TN) was analyzed using the Kjeldahl digestion procedure coupled with distillation and titration [62]. Concentrations of Fe, Mg, Zn, total potassium (TK), and total phosphorus (TP) were obtained after H_2_SO_4_–HClO_4_ digestion, followed by elemental determination with an inductively coupled plasma optical emission spectrometer (ICP–OES; Thermo Fisher Scientific, Waltham, MA, USA). Urease (URE) activity was determined by means of indophenol colorimetry, catalase (CAT) activity via potassium permanganate titration, and the activities of β-glucosidase (BG), cellobiohydrolase (CBH), β-N-acetylglucosaminidase (NAG), and alkaline phosphatase (AKP) were quantified using microplate-based fluorometric assays [63].

### 4.4. Soil DNA Extraction and PCR Amplification

Genomic DNA was isolated from 0.5 g of fresh soil using the E.Z.N.A.^®^ Soil DNA Kit (Omega Bio-tek, Norcross, GA, USA) in accordance with the supplier’s instructions. The integrity of the extracted DNA was verified by 1.0% agarose gel electrophoresis, and its concentration was determined spectrophotometrically with a NanoDrop 2000 instrument (Thermo Fisher Scientific, Waltham, MA, USA). All DNA extracts were preserved at −80 °C prior to downstream analyses. The internal transcribed spacer (ITS) region of fungi was amplified using the primer pair ITS5 (5′-GGAAGTAAAAGTCGTAACAAGG-3′) and ITS4 (5′-TCCTCCGCTTATTGATATGC-3′). PCR amplification was conducted in a total reaction volume of 25 μL containing PCR buffer, dNTPs, forward and reverse primers, Taq polymerase (250 U), template DNA, and double-distilled H_2_O adjusted to the final volume. Each sample was amplified in triplicate to ensure reproducibility. The amplification program consisted of an initial denaturation step at 98 °C for 3 min, followed by 27 cycles including denaturation (98 °C, 15 s), annealing (50 °C, 30 s), and extension (72 °C, 30 s), with a final extension at 72 °C for 10 min. Amplified products from identical samples were combined and separated on 2% agarose gels. Bands corresponding to the target fragments were excised and purified using the AxPrep DNA Gel Extraction Kit (Axygen Biosciences, Union City, CA, USA). After elution with Tris-HCl buffer, product quality was reconfirmed by means of agarose gel electrophoresis. DNA concentrations were quantified using the QuantiFluor™-ST Fluorescence Quantification System (Promega, Madison, WI, USA). Equimolar amplicons were pooled and subjected to high-throughput sequencing on the Illumina NextSeq 2000 platform (Illumina, San Diego, CA, USA) using paired-end 150 bp (PE150) chemistry at Majorbio Bio-Pharm Technology Co., Ltd. (Shanghai, China).

### 4.5. Bioinformatic Analysis

Following the merger of paired-end reads with FLASH v1.2.11, sequences were quality-filtered using Trimmomatic v0.32 to remove low-quality regions, homopolymers (>6 bp), and ambiguous bases. High-quality sequences were clustered into 97% similarity OTUs with UPARSE v7.1. To minimize the influence of uneven sequencing depth among samples, all samples were rarefied to the same number of sequences prior to diversity and network analyses. Alpha diversity (Richness, Shannon, ACE) and coverage were calculated using Mothur v1.30.1. Taxonomy was assigned with the RDP classifier v2.2 after chimera removal against the UNITE ITS Database v7.0, enabling the calculation of relative abundances. Functional guilds were predicted via FUNGuild v1.0 and only assignments with “probable” and “highly probable” confidence rankings were retained for downstream analyses

### 4.6. Statistical Analysis

Significant differences in soil physicochemical properties, dominant fungal abundance, and alpha diversity indices across plantation types were assessed by means of one-way analysis of variance (ANOVA) using SPSS 23.0 (SPSS Inc., Chicago, IL, USA). Post hoc Tukey’s HSD tests were conducted for multiple comparisons when significant differences were detected (*p* < 0.05). Alpha diversity indices, including Ace, Chao, Coverage, Simpson, Shannon, and Sobs, were calculated using Mothur software (v1.48.0) to assess community richness, evenness, and sequencing depth across samples. Co-occurrence networks of fungal communities were constructed based on Spearman correlation analysis of the top 50 abundant genera. The selection of the top 50 genera was used to reduce noise from rare taxa and improve network stability and interpretability. Correlations with |r| > 0.5 and *p* < 0.05 were considered statistically robust, and *p*-values were adjusted using the false discovery rate (FDR) method to reduce false-positive associations. Network visualization and topological analysis were performed using Gephi (v0.10.1). Box plots visualizing α-diversity indices were generated using Origin 2021 (OriginLab, Northampton, MA, USA). Functional profiling of fungal communities was performed based on the FUNGuild database, with corresponding functional composition plots created to represent trophic modes and guild distributions. Principal coordinate analysis (PCoA) and redundancy analysis (RDA) were conducted using the vegan package in R v4.3.0, with corresponding ordination plots generated to visualize community dissimilarities and environment–factor relationships. Prior to RDA, multicollinearity among environmental variables was assessed using variance inflation factors (VIF), and highly collinear variables (VIF > 10) were removed to ensure model robustness and interpretability.

## 5. Conclusions

Soil physicochemical properties, fungal community structure, and co-occurrence networks differed between monoculture and mixed *Quercus variabilis* plantations. Compared with QV monoculture, the PO plantation was associated with higher SOC, TN, and TP, as well as greater fungal richness, diversity, and saprotrophic abundance. In addition, PO and PPQ plantations exhibited more complex fungal networks with greater connectivity. SOC, TN, TP, SWC, Zn, and BG activity were identified as key factors shaping fungal community composition. Overall, mixed plantations, particularly the PO model, have the potential to improve soil conditions and fungal community structure in the oak-dominated systems of western Henan. However, long-term monitoring is required to confirm the stability of these patterns.

## Figures and Tables

**Table 1:** Soil properties in monoculture and mixed *Q. variabilis* plantations.

Soil Factors	Plantation Types			
	QV	PO	PT	PPQ
BD (g·cm^−3^)	1.38 ± 0.05 a	1.25 ± 0.02 b	1.16 ± 0.06 c	1.14 ± 0.08 c
SWC (%)	21.84 ± 0.98 a	23.48 ± 1.32 a	20.43 ± 1.35 a	22.18 ± 1.61 a
pH	7.24 ± 0.22 a	7.12 ± 0.24 b	7.15 ± 0.11 b	7.16 ± 0.25 b
SOC(g·kg^−1^)	17.74 ± 0.25 b	20.99 ± 0.56 a	18.85 ± 0.21 b	21.19 ± 0.33 a
TN (g·kg^−1^)	1.46 ± 0.03 c	1.59 ± 0.04 b	1.41 ± 0.03 c	1.74 ± 0.07 a
TP (g·kg^−1^)	0.24 ± 0.01 b	0.29 ± 0.02 a	0.23 ± 0.01 b	0.25 ± 0.02 b
TK (mg·kg^−1^)	16.62 ± 0.19 c	18.59 ± 0.48 a	18.27 ± 0.41 a	17.47 ± 0.32 b
Fe (mg·kg^−1^)	90.31 ± 3.78 c	104.59 ± 5.12 b	114.71 ± 12.20 a	95.39 ± 2.80 c
Mg (mg·kg^−1^)	2.64 ± 0.26 b	2.86 ± 0.10 ab	3.16 ± 0.45 a	2.48 ± 0.45 b
Zn (mg·kg^−1^)	0.45 ± 0.01 b	0.53 ± 0.02 a	0.49 ± 0.02 b	0.48 ± 0.03 b
URE (mg·g^−1^·d^−1^)	0.80 ± 0.07 a	0.79 ± 0.04 a	0.77 ± 0.04 a	0.78 ± 0.04 a
CAT (mL·g^−1^·h^−1^)	0.19 ± 0.01 c	0.25 ± 0.01 a	0.21 ± 0.02 b	0.18 ± 0.01 c
BG (nmol·g^−1^·h^−1^)	22.12 ± 0.32 d	35.04 ± 0.56 a	30.62 ± 0.46 b	26.44 ± 0.64 c
CBH (nmol·g^−1^·h^−1^)	18.21 ± 0.36 a	14.99 ± 0.21 c	14.41 ± 0.25 c	16.00 ± 0.48 b
NAG (nmol·g^−1^·h^−1^)	3.33 ± 0.08 d	4.02 ± 0.06 c	5.39 ± 0.12 a	4.78 ± 0.07 b
AKP (nmol·g^−1^·h^−1^)	28.98 ± 0.85 d	39.87 ± 1.07 a	36.94 ± 0.82 b	32.08 ± 0.79 c

Different letters indicate significant differences in the same soil factor among plantation types (*p* < 0.05). QV, *Q. variabilis* monoculture; PO, Mixed *Q. variabilis* and *P. orientalis*; PT, Mixed *Q. variabilis* and *P. tabulaeformis*; PPQ, Mixed *Q. variabilis*, *P. orientalis*, and *P. tabulaeformis.*

**Table 2:** Relative abundances of functional groups of soil fungi in monoculture and mixed *Q. variabilis* plantations.

Trophic Modes	Functional Groups	QV	PO	PT	PPQ
Saprotroph	Undefined Saprotroph	19.11% b	30.57% b	37.32% a	32.05% ab
	Wood Saprotroph	8.54% a	12.25% a	0.11% b	1.20% b
	Dung Saprotroph	8.02% a	1.86% b	0.07% b	1.63% b
	Soil Saprotroph	0.17% b	5.93% a	0.00% b	0.07% b
	Plant Saprotroph–Wood Saprotroph	0.01% b	0.01% b	0.46% b	1.55% a
Pathotroph	Animal Pathogen	7.22% a	0.00% b	0.00% b	2.09% b
	Plant Pathogen	0.01% b	0.48% a	0.01% b	0.03% b
Symbiotroph	Ectomycorrhizal	17.74% c	5.27% c	30.40% b	36.98% a
	Ectomycorrhizal–Orchid Mycorrhizal–Root-Associated Biotroph	0.00% b	0.00% b	8.68% a	0.00% b
Mixotroph	Endophyte–Litter Saprotroph–Soil Saprotroph–Undefined Saprotroph	5.12% a	4.94% a	0.84% b	3.54% a
	Dung Saprotroph–Ericoid Mycorrhizal–Lichenized	0.04% b	2.56% a	0.30% b	0.42% b
	Ectomycorrhizal–Undefined Saprotroph	21.5% a	0.00% c	9.36% b	8.65% b
	Animal Pathogen–Undefined Saprotroph	0.06% b	1.12% a	0.17% b	0.27% b
	Animal Pathogen–Dung Saprotroph–Endophyte–Epiphyte–Plant Saprotroph–Wood Saprotroph	0.78% b	4.03% a	0.03% b	0.26% b
	Ectomycorrhizal–Fungal Parasite–Plant Saprotroph–Wood Saprotroph	2.2% a	0.00% b	0.00% b	0.00% b

Different letters after the percentage numbers indicate significant differences among plantation types (*p* < 0.05).

## Data Availability

The original contributions presented in this study are included in the article. Further inquiries can be directed to the corresponding author.

## Abbreviations

The following abbreviations are used in this manuscript:

SOC	Soil Organic Carbon
TN	Total Nitrogen
TP	Total Phosphorus
TK	Total Potassium
BD	Bulk Density
SWC	Soil Water Content
pH	Soil pH
Fe	Iron
Mg	Magnesium
Zn	Zinc
URE	Urease
CAT	Catalase
BG	β-glucosidase
CBH	Cellobiohydrolase
NAG	β-N-acetylglucosaminidase
AKP	Alkaline Phosphatase
ITS	Internal Transcribed Spacer
PCR	Polymerase Chain Reaction
OTUs	Operational Taxonomic Units
PCoA	Principal Coordinate Analysis
RDA	Redundancy Analysis
ANOVA	Analysis of Variance
Ace	Abundance-based Coverage Estimator
Chao	Chao1 Richness Estimator
Sobs	Observed Species
QV	*Q. variabilis* monoculture plantation
PO	Mixed *Q. variabilis* and *P. orientalis* plantation
PT	Mixed *Q. variabilis* and *P. tabulaeformis* plantation
PPQ	Mixed *Q. variabilis*, *P. orientalis*, and *P. tabulaeformis* plantation

## References

[1] Berendsen R.L., Pieterse C.M.J., Bakker P.A.H.M. (2012). The rhizosphere microbiome and plant health. Trends Plant Sci..

[2] Ma X.D., Zou J.L., Wu J.Y. (2024). Responses of soil bacterial and fungal community structure and functions to different plant species in a mixed forest plantation in a semi-arid region of China. Appl. Soil Ecol..

[3] Saleem M., Hu J., Jousset A. (2019). More than the sum of its parts: Microbiome biodiversity as a diver of plant growth and soil health. Annu. Rev. Ecol. Evol..

[4] Xu Y.X., Ren S.Q., Liang Y.F., Du A., Li C., Wang Z.C., Zhu W.K., Wu L.C. (2021). Soil nutrient supply and tree species drive changes in soil microbial communities during the transformation of a multi-generation Eucalyptus plantation. Appl. Soil Ecol..

[5] Ni H.J., Su W.H., Fan S.H., Chu H.Y. (2021). Effects of intensive management practices on rhizosphere soil properties, root growth, and nutrient uptake in Moso bamboo plantations in subtropical China. For. Ecol. Manag..

[6] Xu Z.Y., Hu Z.H., Jiao S., Bell S.M., Xu Q., Ma L.L., Chen J. (2023). Depth-dependent effects of tree species identity on soil microbial community characteristics and multifunctionality. Sci. Total Environ..

[7] Li W.Q., Huang Y.X., Chen F.S., Liu Y.Q., Lin X.F., Zong Y.Y., Wu G.Y., Yu Z.R., Fang X.M. (2021). Mixing with broad-leaved trees shapes the rhizosphere soil fungal communities of coniferous tree species in subtropical forests. For. Ecol. Manag..

[8] Baldrian P. (2017). Forest microbiome: Diversity, complexity and dynamics. Fems Microbiol. Rev..

[9] Zhao M., Sun Y.R., Liu S.H., Li Y.C., Chen Y.M. (2024). Effects of stand density on the structure of soil microbial functional groups in *Robinia pseudoacacia* plantations in the hilly and gully region of the Loess Plateau, China. Sci. Total Environ..

[10] Frac M., Hannula S.E., Belka M., Jedryczka M. (2018). Fungal biodiversity and their role in soil health. Front. Microbiol..

[11] Wang C.W., Ma L.N., Zuo X., Ye X.H., Wang R.Z., Huang Z.Y., Liu G.F., Cornelissen J.H.C. (2022). Plant diversity has stronger linkage with soil fungal diversity than with bacterial diversity across grasslands of northern China. Glob. Ecol. Biogeogr..

[12] Smith S., Read D.J. (2008). Mycorrhizal Symbiosis.

[13] Qiu Y.P., Jiang Y., Guo L.J., Zhang L., Burkey K.O., Zobel R.W., Reberg-Horton S.C., Shew H.D., Hui S.J. (2019). Shifts in the composition and activities of denitrifiers dominate CO_2_ stimulation of N_2_O emissions. Environ. Sci. Technol..

[14] van der Heijden M.G., Bardgett R.D., van Straalen N.M. (2008). The unseen majority: Soil microbes as drivers of plant diversity and productivity in terrestrial ecosystems. Ecol. Lett..

[15] Adnan M., Islam W., Gang L., Chen H.Y.H. (2022). Advanced research tools for fungal diversity and its impact on forest ecosystem. Environ. Sci. Pollut. Res..

[16] Li Z.B., Song Z., Qiao R.Y., Xu M.Z., Wu X.Y., Chen Y.F., Zhang P.D., Ding C.J., Chen Y.L., Guo H. (2024). The beneficial and pathogenic flora, environmental drivers, and community assembly mechanism of perennial poplar plantation. Plant Soil.

[17] Monkai J., Hyde K.D., Xu J.C., Mortimer P.E. (2017). Diversity and ecology of soil fungal communities in rubber plantations. Fungal Biol. Rev..

[18] Karimi B., Maron P.A., Boure N.C.P., Bernard N., Gilbert D., Ranjard L. (2017). Microbial diversity and ecological networks as indicators of environmental quality. Environ. Chem. Lett..

[19] Xu Y.X., Li C., Zhu Y.L., Wang Z.C., Zhu W.K., Wu L.C., Du A.P. (2022). The shifts in soil microbial community and association network induced by successive planting of Eucalyptus plantations. For. Ecol. Manag..

[20] Li Y.G., Han C., Dong X.X., Sun S., Zhao C.M. (2022). Soil microbial communities of dryland legume plantations are more complex than non-legumes. Sci. Total Environ..

[21] Wang Y.X., Zhu Y., Meng S., Wang S.K., Yang F.C., Qin F.C., Lu J.K. (2025). Linkages between the soil microbial network and nutrient cycling along a subtropical plantation chronosequence. Plant Soil.

[22] Vacher C., Tamaddoni-Nezhad A., Kamenova S., Peyrard N., Moalic Y., Sabbadin R., Schwaller L., Chiquet J., Smith M.A., Vallance J. (2016). Learning ecological networks from next-generation sequencing data. Adv. Ecol. Res..

[23] Chen D.M., Zhang X.X., Kang H.Z., Sun X., Yin S., Du H.M., Yamanaka N., Gapare W., Wu H.X., Liu C.J. (2012). Phylogeography of *Quercus variabilis* based on chloroplast DNA sequence in East Asia: Multiple glacial refugia and mainland-migrated island populations. PLoS ONE.

[24] Yuan J., Sun N.X., Du H.M., Yin S., Kang H.Z., Umair M., Liu C.J. (2020). Roles of metabolic regulation in developing Quercus variabilis acorns at contrasting geologically-derived phosphorus sites in subtropical China. BMC Plant Biol..

[25] Liu Y.C., Tian H.M., Liu S.R., Li G.Y., Hu X.J. (2022). Asymmetric effects between tree and understorey litters on mixed litter decomposition in temperate *Quercus variabilis* forest. Sci. Total Environ..

[26] Wang J., Liu C., Shao X.L., Song Y.T., Wang X. (2025). Influence of tree species composition on leaf and soil properties and soil enzyme activity in mixed and pure Oak (*Quercus variabilis*) stands. Forests.

[27] Gong C., Tan Q.Y., Liu G.B., Xu M.X. (2021). Mixed-species plantations enhance soil carbon stocks on the loess plateau of China. Plant Soil.

[28] Wongprom J., Jumwong N., Sangvisitpirom P., Diloksumpun S., Thaopimai L. (2025). Growth, productivity, and nutrient return of a mixed plantation of fast-growing *Eucalyptus hybrid* and *Acacia auriculiformis* trees in Thailand. Forests.

[29] Yu Z.J., Wang K.B., Li J.W., Shangguan Z.P., Deng L. (2022). Mixed plantations have more soil carbon sequestration benefits than pure plantations in China. For. Ecol. Manag..

[30] Guan S.Y., Lu Y.C., Liu X.Z. (2022). Evaluation of multiple forest service based on the integration of stand structural attributes in mixed oak forests. Sustainablity.

[31] Wu X., Niu Y.B., Xun M.Y., Jin J.Y., Tang Y.K., Chen Y.M. (2021). Soil carbon, nitrogen, and phosphorus storages and their stoichiometry due to mixed afforestation with *Hippophae rhamnoides* in the Loess Hilly Region, China. Forests.

[32] Prada-Salcedo L.D., Goldmann K., Heintz-Buschart A., Reitz T., Wambsganss J., Bauhus J., Buscot F. (2021). Fungal guilds and soil functionality respond to tree community traits rather than to tree diversity in European forests. Mol. Ecol..

[33] Sun R.B., Chen Y., Han W.X., Dong W.X., Zhang Y.M., Hu C.S., Liu B.B., Wang F.H. (2020). Different contribution of species sorting and exogenous species immigration from manure to soil fungal diversity and community assemblage under long-term fertilization. Soil Biol. Biochem..

[34] Cheng X.R., Zhang Y.L., Xu H.D. (2024). Conversion of monoculture plantation to two-aged mixed plantation enhances soil organic carbon via increased microbial residue carbon accrual. Catena.

[35] Lawson S.S., Frene J.P., Sue N.D.L. (2024). Fungal footprints: Soil fungal communities in black walnut and red oak forests. Microorganism.

[36] Yang Y., Cheng H., Dou Y.X., An S.S. (2020). Plant and soil traits driving soil fungal community due to tree plantation on the Loess Plateau. Sci. Total Environ..

[37] Curlevski N.J.A., Xu Z.H., Anderson I.C., Cairney J.W.G. (2010). Converting Australian tropical rainforest to native Araucariaceae plantations alters soil fungal communities. Soil Biol. Biochem..

[38] Manici L.M., Caputo F., Fornasier F., Paletto A., Ceotto E., De Meo I. (2024). Ascomycota and Basidiomycota fungal phyla as indicators of land use efficiency for soil organic carbon accrual with woody plantations. Ecol. Indic..

[39] Frossard A., Gerull L., Mutz M., Gessner M.O. (2013). Litter supply as a driver of microbial activity and community structure on decomposing leaves: A test in experimental streams. Appl. Environ. Microbiol..

[40] Peng Y., Holmstrup M., Schmidt I.K., Bachega L.R., Schelfhout S., Zheng H.F., Hedenec P., Yue K., Vesterdal L. (2022). Tree species identity is the predominant modulator of the effects of soil fauna on leaf litter decomposition. For. Ecol. Manag..

[41] Liu X., Zhao P.S., Gao G.L., Liu X., Zhao P.S., Gao G.L., Ren Y., Ding G.D., Zhang Y. (2024). Niche differentiation shapes the community assembly of fungi associated with evergreen trees in the Horqin desert. Appl. Soil Ecol..

[42] Beugnon R., Bu W.S., Bruelheide H., Davrinche A., Du J.Q., Haider S., Kunz M., von Oheimb G., Perles-Garcia M.D., Saadani M. (2023). Abiotic and biotic drivers of tree trait effects on soil microbial biomass and soil carbon concentration. Ecol. Monogr..

[43] Lyu M.K., Xie J.S., Giardin C.P., Vadeboncoeur M.A., Feng X., Wang M.H., Ukonmaanaho L., Lin T.C., Kuzyakov Y., Yang Y.S. (2019). Understory ferns alter soil carbon chemistry and increase carbon storage during reforestation with native pine on previously degraded sites. Soil Biol. Biochem..

[44] Anderson H.M., Cagle G.A., Majumder E.L.W., Silva E., Dawson J., Simon P., Freedman Z.B. (2024). Root exudation and rhizosphere microbial assembly are influenced by novel plant trait diversity in carrot genotypes. Soil Biol. Biochem..

[45] Broeckling C.D., Broz A.K., Bergelson J., Manter D.K., Vivanco J.M. (2008). Root exudates regulate soil fungal community composition and diversity. Appl. Environ. Microbiol..

[46] Baldrian P., Vetrovsky T., Lepinay C., Kohout P. (2022). High-throughput sequencing view on the magnitude of global fungal diversity. Fungal Divers..

[47] Yang T., Adams J.M., Shi Y., He J.S., Jing X., Chen L.T., Tedersoo L., Chu H.Y. (2017). Soil fungal diversity in natural grasslands of the Tibetan Plateau: Associations with plant diversity and productivity. New Phytol..

[48] Gardner T.G., Frene J.P., Lawson S.S., Sue N.D.L., Handy J., Crawford R.H. (2023). The impact of tree species on microbial community structure and soil function on forest plantations in the Central Hardwoods Region (CHR). Forests.

[49] Qiao L., Guan Z.Z., Ren F.F., Ma T.X. (2025). Comparative analysis of rhizosphere microbial communities in monoculture and mixed oak-pine forests: Structural and functional insights. Front. Microbiol..

[50] Shen F.Y., Liu N., Wang Y.J., Liu H.F., Jia H.K., Yang L.X. (2023). The effects of Korean Pine and Manchurian Walnut monocultures and mixed plantations on soil fungal, and bacterial communities. Forests.

[51] Zhao H., Zhang F.B., Wang Y., Wang J.M., Li J.W., Zhang Z.X. (2023). Variation and drivers of soil fungal and functional groups among different forest types in warm temperate secondary forests. Glob. Ecol. Conserv..

[52] Pii Y., Mimmo T., Tomasi N., Terzano R., Cesco S., Crecchio C. (2015). Microbial interactions in the rhizosphere: Beneficial influences of plant growth-promoting rhizobacteria on nutrient acquisition process. A review. Biol. Fertil. Soils.

[53] Wang Y.H., Hong L., Li J.J., Zhang Q.X., Wang A.Q., Lin S.X., Hu M.Y., Chen Y.L., Lin W.X., Wang H.B. (2024). Analysis of growth inhibition of continuously planted Casuarina equisetifolia in relation to characteristic soil microbial functions and nutrient cycling. Appl. Soil Ecol..

[54] Suetsugu K., Matsuoka S., Shutoh K., Okada H., Taketomi S., Onimaru K., Tanabe A.S., Yamanaka H. (2021). Mycorrhizal communities of two closely related species, *Pyrola subaphylla* and *P. japonica*, with contrasting degrees of mycoheterotrophy in a sympatric habitat. Mycorrhiza.

[55] Ma B., Wang H.Z., Dsouza M., Lou J., He Y., Dai Z.M., Brookes P.C., Xu J.M., Gilbert J.A. (2016). Geographic patterns of co-occurrence network topological features for soil microbiota at continental scale in eastern China. ISME J..

[56] Fierer N., Wood S.A., de Mesquita C.P.B. (2021). How microbes can, and cannot, be used to assess soil health. Soil Biol. Biochem..

[57] Schloter M., Nannipieri P., Sørensen S.J., van Elsas J.D. (2018). Microbial indicators for soil quality. Biol. Fertil. Soils..

[58] Tkacz A., Cheema J., Chandra G., Grant A., Poole P.S. (2015). Stability and succession of the rhizosphere microbiota depends upon plant type and soil composition. ISME J..

[59] Lozano Y.M., Aguilar-Trigueros C.A., Roy J., Rillig M.C. (2021). Drought induces shifts in soil fungal communities that can be linked to root traits across 24 plant species. New Phytol..

[60] Kutos S., Barnes E.M., Lewis J.D. (2023). Soil fungal communities vary more with soil characteristics than tree diversity at a local scale. Can. J. For. Res..

[61] Nelson D.W., Sommers L.E. (1982). Total carbon, organic carbon and organic matter. Methods of Soil Analysis, Part 2.

[62] Jackson M.L. (1973). Soil Chemical Analysis.

[63] DeForest J.L. (2009). The influence of time, storage temperature, and substrate age on potential soil enzyme activity in acidic forest soils using MUB-linked substrates and L-DOPA. Soil Biol. Biochem..

